# A novel leaf-rolling chironomid, *Eukiefferiella endobryonia* sp. nov. (Diptera, Chironomidae, Orthocladiinae), highlights the diversity of underwater chironomid tube structures

**DOI:** 10.3897/zookeys.906.47834

**Published:** 2020-01-22

**Authors:** Yume Imada

**Affiliations:** 1 Graduate School of Science and Engineering, Ehime University, 2-5 Bunkyo-cho, Matsuyama, Ehime 790-8577, Japan Ehime University Ehime Japan

**Keywords:** bryophytivore, freshwater, Orthocladiinae, tubicolous, *
Eukiefferiella
*

## Abstract

The non-biting midges, Chironomidae (Diptera), are dominant components of most freshwater ecosystems. Many chironomids construct tubes or cases as larvae out of various materials bound together with silk. The structures of tubes show a wide range of variation, and some are morphologically comparable to those of caddisflies. Herein a new species is described, *Eukiefferiella endobryonia***sp. nov.**, which exhibits a very unusual behavior in which it constructs tubes from aquatic mosses. This species’ fourth-instar larvae construct their cases exclusively from the leaves of *Fontinalis* mosses (Hypnales: Fontinalaceae) and exhibit a stereotyped behavior in which they remain attached to the apical shoot of the moss stem. The larvae then pupate within the case. The case of *E. endobryonia***sp. nov.** represents one of only a few examples of chironomid tubes made exclusively out of plants. Based on the species delimitation analyses using the partial COI sequences, together with some morphological and behavioral characteristics, this species is hypothesized to be a member of *devonica* group, and especially may have a close affinity to *E. dittmari* (Lehman). A provisional typology for the diversity of chironomid tube structures is provided, with a summary of different tube structures, which can be used for future research.

## Introduction

Many aquatic animals build biogenic structures, such as burrows, tubes, and cases (Chamberlain 1975). Construction behavior has evolved in a taxonomically diverse array of animals, including Protozoa, Mollusca, Annelida, Polychaeta, Crustacea, Echinodermata, fishes, and Nematoda (Nehring et al. 1990; Dudgeon 1994; Charbonneau and Hare 1998; Nehring 1993; Merz 2015). Dwelling tubes can be preserved as ichnofossils and provide evidence for activities by organisms over geological time (Chamberlain 1975; Gall 1983; Minter et al. 2016). In the freshwater realm, three insect orders contain notable numbers of species with such tube construction behaviors (Wallace and Merritt 1980): Trichoptera, Diptera (family Chironomidae), and Ephemeroptera (family Polymitarcyidae). Tube morphology and ultrastructure vary significantly among taxa, and these tubes may serve various functions (Dudgeon 1994).

Chironomidae is a diverse nematocerous family of Diptera, to which ca. 7290 described species belong (Pape et al. 2011; Courtney et al. 2017). Larval chironomids inhabit a broad spectrum of habitats, especially in permanent and temporal freshwater environments ranging from running and standing water, to madicolous zones, temporary pools, and phytotelmata; a substantial number of species also occur in terrestrial habitats (Oliver 1971; Pinder 1995). Their modes of feeding are also diverse (Thienemann 1954). They have been classified as belonging to most functional feeding groups (Cummins 1974), including collector-gatherers, collector-filterers, scrapers, shredders, engulfers, and piercers (predators and parasites) (Berg 1995).

Most larvae of Chironomidae construct dwelling tubes or cases by combining various particles together with silk (Oliver 1971) that they secrete from their labial gland (Sehnal and Sutherland 2008). In many cases, chironomid tubes are soft, flexible, and cryptic in sediments (Heckman 2018). Several functions have been hypothesized for the tubicolous habit of these species, including improved respiration (Walshe 1950; Kon and Hidaka 1983; Stief et al. 2005), feeding (Walshe 1947, 1951), anti-predator defense (Hershey 1987), and protection against physiological stress (e.g., toxicity and desiccation) (Hinton 1951; Kikawada et al. 2005; Vedamanikam and Shazili 2009). Tube-making chironomids can be important ecosystem engineers (Lawton and Jones 1995). Tube-dwelling chironomids can be pioneer species, as they often colonize newly submerged substrata first and in greater numbers than other colonists (Nilsen and Larimore 1973). Tube-dwelling, deposit-feeding chironomids play important roles in the bioturbation of organic detritus (Dudgeon 1994; Svensson and Leonardson 1996; Chapin 2011). Consequently, they impact the physical properties of sediments and drive biogeochemical processes in lake ecosystems (Ólafsson and Paterson 2004; Hölker et al. 2015). The presence and feeding activities of chironomids also have impacts on the structures of meiofaunal and protozoan communities (Ptatscheck et al. 2017; Webert et al. 2017). Additionally, tube structures, by themselves, can exert considerable effects on the periphytic diatom flora living on river rocks (Pringle 1985; Hershey et al. 1988; Herren et al. 2017).

In lotic habitats, aquatic mosses harbor various benthic invertebrates (Suren 1993). In many stream habitats, Chironomidae can be abundant on clumps of aquatic fountain mosses, *Fontinalis* spp. (Linhart et al. 2002; Vlčková et al. 2002; Bogut et al. 2009). Aquatic bryophytes can interact with aquatic arthropods by providing them with space and shelter from predators (Stream Bryophyte Group 1999). However, it is unclear whether aquatic bryophytes are an important food source for invertebrates (Winterbourn et al. 1986; McWilliam-Hughes et al. 2009), or not (Bunn et al. 1989; Suren and Winterbourn 1991). The inhibition of invertebrate feeding on bryophytes is often explained as likely resulting from the low nutritional value and presence of secondary chemicals in aquatic mosses (Parker et al. 2007).

While searching for arthropods that interact with aquatic bryophytes in North America, I discovered chironomid larvae that were notably distinct from other tubicolous chironomids due to their unique tube-constructing behavior. Specifically, the fourth-instar larvae of these chironomids make cases exclusively using the leaves of *Fontinalis* mosses. Although a number of chironomid larvae were found attached to moss shoots, some larvae could be clearly distinguished by their construction behavior at the shoot tips of *Fontinalis* mosses. This species turned out to be a new species belonging to the genus *Eukiefferiella* Thienemann (Orthocladiinae). The life history of this species was clarified with the aid of DNA barcoding, and a description of it is given herein, including an account of its larval tube construction behavior. As the taxonomy of *Eukiefferiella* can be problematic, the genetic differentiation of the new species in comparison to some congeners which are hypothetically closely related is estimated using methods for delimitation of species. Additionally, a provisional typology of chironomid tube morphology is provided, to highlight the diverse morphology of tube structure among chironomids.

## Materials and methods

### Study sites

Chironomids were collected during 24–27 February and on 10 November in 2018 in a stream connected to Mountain Lake, Virginia, USA. Mountain Lake is an oligotrophic lake that is located at an elevation of 1181 m above sea level near the summit of Salt Pond Mountain, and is the only natural lake of substantial size in the unglaciated part of the Southern Appalachian Mountain Range (Sharp 1933). Mountain Lake possesses an unusually high diversity of aquatic plants and invertebrates (reviewed by Parker 2003). Two species of *Fontinalis* have been recorded from the lake itself and the brooks in its vicinity, *F. dalecarlica* B. S. G. and *F. novae-angliae* Sull. (Studler 1982). *F. dalecarlica* is common in gently flowing water bodies, including in the brook that I sampled. Additional samples were collected during 4–9 April and 11–13 November in 2018 in a flowing-water stream, Rowans River, along Sparks Lane in Cades Cove, in the northwest part of the Great Smoky Mountains National Park (GRSM), Tennessee, USA, ca. 430 km away from Mountain Lake. *Fontinalis novae-angliae* with rigid stems and concave leaves occur from the streambed. Samples were collected in GRSM under research permit GRSM-2017-SCI-2389.

### Collections, rearing, and observations

Insects were searched for underwater in the sampled streams and brooks and were collected together with the host plants occurring in their habitats. At Mountain Lake Biological Station, the clumps of mosses were detangled from detritus, and sediments were washed out of them. The larvae were placed in small plastic cases and observed with a microscope. The plastic chambers were constantly cooled with a refrigerant to keep their temperature in the range between 10–22 °C. Fourth-instar larvae were observed for 27 h in total, between 08:00 hrs and 18:00 hrs during 4–25 March 2018. Rearing and observations of chironomid larvae were performed at the National Museum of Natural History, Smithsonian Institution.

### Molecular analyses

To compare and differentiate the chironomids of different stages and sexes occurring at the study sites, their partial COI (cytochrome c oxidase subunit I) gene sequences were determined. Total genomic DNA was extracted from 23 specimens at different stages, including adults (from a single adult leg or abdomen), larvae (two or three abdominal segments), and pupae (the whole abdomen) or pupal exuviae, using a NucleoSpin Tissue kit (Macherey-Nagel, Düren, Germany) and following the protocol provided by the manufacturer, with some modifications. The protocol was modified as follows: (i) tissue was digested for 48 h at 58 °C; (ii) after digestion with proteinase K, tissues were removed, washed in distilled water and used for morphological assessments; and (iii) the final elution volume was 30 μL. The primer pair used for the COI region consisted of primers 911 and 912 of Folmer et al. (1994), as used in some previous DNA barcoding and phylogenetic studies of Chironomidae (e.g., Guryev et al. 2001; Stur and Ekrem 2011; Cranston et al. 2012). Amplifications of the COI region were performed in a thermocycler, with an initial denaturation step of 94 °C for 4 min, followed by 40 cycles of 94 °C for 45 s, 55 °C for 45 s, and 72 °C for 1 min, and one cycle at 72 °C for 10 min. Amplification products were purified with ExoSAP-IT, according to the manufacturer’s instructions.

Direct sequencing of polymerase chain reaction (PCR) products was performed using the ABI Big Dye Terminator 3.1 cycle sequencing kit (Applied Biosystems, Lennik, Belgium) while following the manufacturer’s instructions and was carried out in an ABI 3130 Capillary Electrophoresis Genetic analyzer. Both DNA strands were sequenced. Sequences were deposited in the GenBank database (Table 1).

**Table 1.** Specimen and collection information used for the DNA barcoding analysis. Summary of specimens used for the COI analyses. Species names identified by morphology.

Species	Voucher ID	Accession number	Collection locality	Coordinates	Reference
*Cardiocladius capucinus*	ATNA466	HM421556.1	Norway: Rondane National Park	61.9935N, 9.80343E	GenBank
*Cardiocladius fuscus*	NIESH0714	LC329044.1	Japan: Nagano, River Chikuma	–	GenBank
*E. claripennis*	ATNA247	HM421358.1	Norway: Rondane National Park	61.9819N, 9.80454E	GenBank
	ATNA260	HM421370.1	Norway: Rondane National Park	61.9835N, 9.80384E	GenBank
	ATNA354	HM421455.1	Norway: Rondane National Park	61.9819N, 9.80454E	GenBank
	Finnmark412	JN275486.1	Norway: Fálleveaijohka	69.6779N, 30.4494E	GenBank
	BIOUG05490-D09	KR175110.1	Canada: Ontario, Rouge National Urban Park	43.8223N, 79.1897W	GenBank
	BIOUG10589-G08	KR276839.1	Canada: Gros Morne National Park	49.5686N, 57.8302W	GenBank
	BIOUG09943-A01	KR276908.1	Canada: Quebec: Forillon National Park	48.857N, 64.376W	GenBank
	BIOUG09308-C08	KR282929.1	Canada: Ontario: Georgian Bay Islands National Park	44.7418N, 79.8501W	GenBank
	BIOUG09943-C02	KR283193.1	Canada: Quebec: Forillon National Park	48.857N, 64.376W	GenBank
*E. devonica*	ATNA239	HM421351.1	Norway: Rondane National Park	61.9819N, 9.80454E	GenBank
	ATNA241	HM421353.1	Norway: Rondane National Park	61.9819N, 9.80454E	GenBank
	ATNA246	HM421357.1	Norway: Rondane National Park	61.9819N, 9.80454E	GenBank
	ATNA499	HQ551492.1	Norway: Rondane National Park	61.9819N, 9.80454E	GenBank
*E. dittmari*	Finnmark194	JF870841.1	Norway: Masi	69.4482N, 23.7576E	GenBank
*E. endobryonia* sp. nov.	YI-CR-001	LC505506	USA: TN: Great Smoky Mountains National Park	35.600894N, 83.794004W	This study
	YI-CR-006	LC505507	USA: TN: Great Smoky Mountains National Park	35.600894N, 83.794004W	This study
	YI-CR-008	LC505508	USA: TN: Great Smoky Mountains National Park	35.600894N, 83.794004W	This study
	YI-CR-009	LC505509	USA: VA: Mountain Lake	37.357627N, 80.534448W	This study
	YI-CR-015	LC505510	USA: VA: Mountain Lake	37.357627N, 80.534448W	This study
*E. ilkleyensis*	ATNA348	HM421450.1	Norway: Rondane National Park	61.9819N, 9.80454E	GenBank
	ATNA497	HQ551490.1	Norway: Rondane National Park	61.9819N, 9.80454E	GenBank
	ATNA498	HQ551491.1	Norway: Rondane National Park	61.9819N, 9.80454E	GenBank
	ATNA512	HQ551503.1	Norway: Rondane National Park	61.9819N, 9.80454E	GenBank
	ATNA513	HQ551504.1	Norway: Rondane National Park	61.9819N, 9.80454E	GenBank
*E. minor*	Finnmark570	JF870931.1	Norway: Rafsbotn	70.0137N, 23.5547E	GenBank
*E.* sp.	BIOUG01648-H02	KR660601.1	Canada: Ontario: Elizabethtown-Kitley	44.618N, 75.775W	GenBank

### Phylogenetic analyses

Sequence trace files were edited with 4Peaks v. 1.8 (by A. Griekspoor and Tom Groothuis, nucleobytes.com). Nucleotide sequences were aligned with Clustal W implemented in MEGA 7 (Kumar et al. 2016). To evaluate if the new species is phylogenetically exclusive among hypothetically closely related species (particularly the species in ‘*devonica*’ group, as discussed later) and to assess the intra- and interspecific genetic distances, the species delimitation plug-in in the software Geneious Prime 2019.2.3 (www.geneious.com) was used (Masters et al. 2011; Kearse et al. 2012).

The COI sequences of a rich record of *Eukiefferiella* species were found in GenBank with 1052 fragment sequences (accessed on October 10^th^, 2019), although the sequence data identified at the species level were available only for five species (i.e., *E. devonica* (Edw.), *E. ilkleyensis* (Edw.), *E. claripennis* (Lundbeck), *E. minor* (Edw.), *E. dittmari* (Lehman)). In the dataset, 23 sequence data representing five species were included (Table 1), as well as five sequences obtained in this study. Phylogenetic trees were inferred by Bayesian inference (BI). Trees were rooted with two species of the genus *Cardiocladius* (i.e., *C. capucinus* (Zetterstedt), *C. fuscus* Kieff.). Evolutionary model was selected with MrModeltest v. 4.0b10 (Nylander 2004). The best fitting models were chosen with the Akaike Information Criterion (Akaike 1973). For the COI dataset, GTR + Gamma model was selected and used for the following Bayesian phylogenetic analysis. BI trees were constructed with MrBayes v.3.2.6 (Huelsenbeck and Ronquist 2001), using the plug-in of Geneious Prime, based on a cold chain and four heated chains with T = 0.2, running for 1,100,000 generations with a sample frequency of 200. The first 100,000 trees were discarded and the remaining trees were used to build a consensus tree, with estimated Bayesian posterior probabilities (PP). At the point of burn-in, the chains had all converged to a stable standard deviation of split frequencies lower than 0.01.

The species delimitation plug-in in the Geneious Prime 2019.2.3 (Kearse et al. 2012; Masters et al. 2011) was used (1) to measure the phylogenetic support of new species described herein, and (2) to evaluate the genetic differentiation among and within species, among other congeners which are hypothetically closely related to this species. For these purposes, Rosenberg’s P_AB_ (Rosenberg 2007) Rodrigo’s P (Randomly Distinct) (Rodrigo et al. 2008) were calculated. Rosenberg’s P_AB_ is a test for taxonomic distinctiveness of a clade based on the null hypothesis that monophyly is a chance outcome of random branching. Rodrigo’s P(Randomly Distinct) (‘Rodrigo’s P (RD)’) is the probability that a focal clade has the observed degree of distinctiveness (i.e., the ratio between the distance from a species-defining node to the tips of the tree, and the distance from that same node to its immediate ancestor) due to random coalescent processes (Rodrigo et al. 2008). Focal groups with values between 0.05 and 1 represent groups that have branching events that would be expected under the coalescent model in a Wright-Fishe population and a strict molecular clock. Additionally, six statistics which are useful to species delimitation were presented, along with Rosenberg’s P_AB_ and Rodrigo’s P (RD): the average pairwise tree distance, among members of the focal species/populations (‘D Intra’) and between the members of the focal species and members of the next closest species (‘D Inter’); as a measure of genetic differences between the focal species and its closest neighboring species, the ratio of D Intra to D Inter (‘Intra/Inter’); as the measures for evaluating diagnosability of each species/population, the mean probability of making a correct identification of a hypothetical sample of the focal species using placement on a tree under two different criteria, ‘P ID(Strict)’ (the sample must fall within the species clade) and ‘P ID(Liberal)’ (the sample is allowed to fall sister to or within a species clade); the mean distance between the most recent common ancestor of a species and its members (‘Av(MRCA)’). Accession number, voucher ID, and information on the localities for each specimen are shown in Table 1.

### Morphological analyses

All specimens of adult abdomens, pupae, and larvae were digested with proteinase K, which made it relatively easy to examine the specimens morphologically. When necessary, the apical portion of the adult abdomen was macerated with warm (ca. 90 °C) 5% KOH and rinsed with distilled water. Each body part sample was mounted on a microscopic slide with Euparal.

The terminology of morphological features used herein followed Sæther (1971, 1977, 1980). The antennal ratio (AR), leg ratios (LR, BV, SV), wing ratio (L/WR) and hypopygium ratio (HR), and other morphological features were measured for adult male specimens following Sæther (1968), Schlee (1966) and Soponis (1977). Abbreviations that are used in this work:

**AR** antennal ratio: length of last flagellomere / length of remaining flagellomeres;

**LR** leg ratio: length of first tarsal segment/ length of tibia;

**BV** “Beinverhältnis”: length of femur, tibia plus first tarsal segment/ length of tarsal segments 2–5;

**SV** length of femur plus tibia/ length of tarsal segments 1–3;

**L/WR** wing length/ wing width ratio;

**HR**, hypopygium ratio: length of gonocoxite/length of gonostylus.

The type specimens are deposited in **NMNH** (National Museum of Natural History, Washington DC, USA). For each specimen, voucher ID is given as ‘YI-CR-##’.

## Taxonomy

### Eukiefferiella endobryonia sp. nov.

Taxon classificationAnimaliaDipteraChironomidae

9358422A-D137-5BA3-8BC4-629764467C1F

http://zoobank.org/2EFD6644-44B9-4CF2-8C5F-6E2523DE6CF6

Figs 1 , 2

#### Diagnosis.

Adult male with squama with few (two or three) setae; gonostylus with crista dorsalis; hind tibial comb and tibial spurs reduced, outer spur absent. Pupa lacks precorneal setae and respiratory horns; three anal macrosetae consisting of two thinner inner macrosetae and a normal outer macroseta. Larval body setae short; seta interna with five branches deeply divided to the base; mentum with four pairs of lateral teeth and single, wide, truncate median tooth.

#### Material examined.

***Holotype***: USA, VA • 1 adult male (YI-CR-013); Mountain Lake (37.357627 N 80.534448 W); 24-II-2018 (as larva); Y. Imada leg; emerged as adult on 12-III-2018; NMNH.

***Paratypes***: USA, VA • 2 adult males (YI-CR-009, YI-CR-016) and 3 adult females (YI-CR-010, YI-CR-011, YI-CR-015); Mountain Lake (37.357627N 80.534448W); 24-II-2018 (as larvae); Y. Imada leg; emerged as adults between 12-III-2018 and 28-IV-2018; NMNH.

#### Other material.

USA, TN • 2 female pupae (YI-CR-001, YI-CR-002), 2 larvae (YI-CR-006, YI-CR-007); Sparks Lane (35.600894N, 83.794004W); 13-XI-2018 (as larvae); Y. Imada leg; NMNH; VA • 1 male pupa (YI-CR-012), 2 female pupa (YI-CR-005, YI-CR-015), 1 pupal exuviae (no voucher), 4 larvae (YI-CR-003, YI-CR-023, YI-CR-024, YI-CR-025); Mountain Lake (37.357627N, 80.534448W); 9-XI-2018; Y. Imada leg, NMNH.

**Figure 1.**
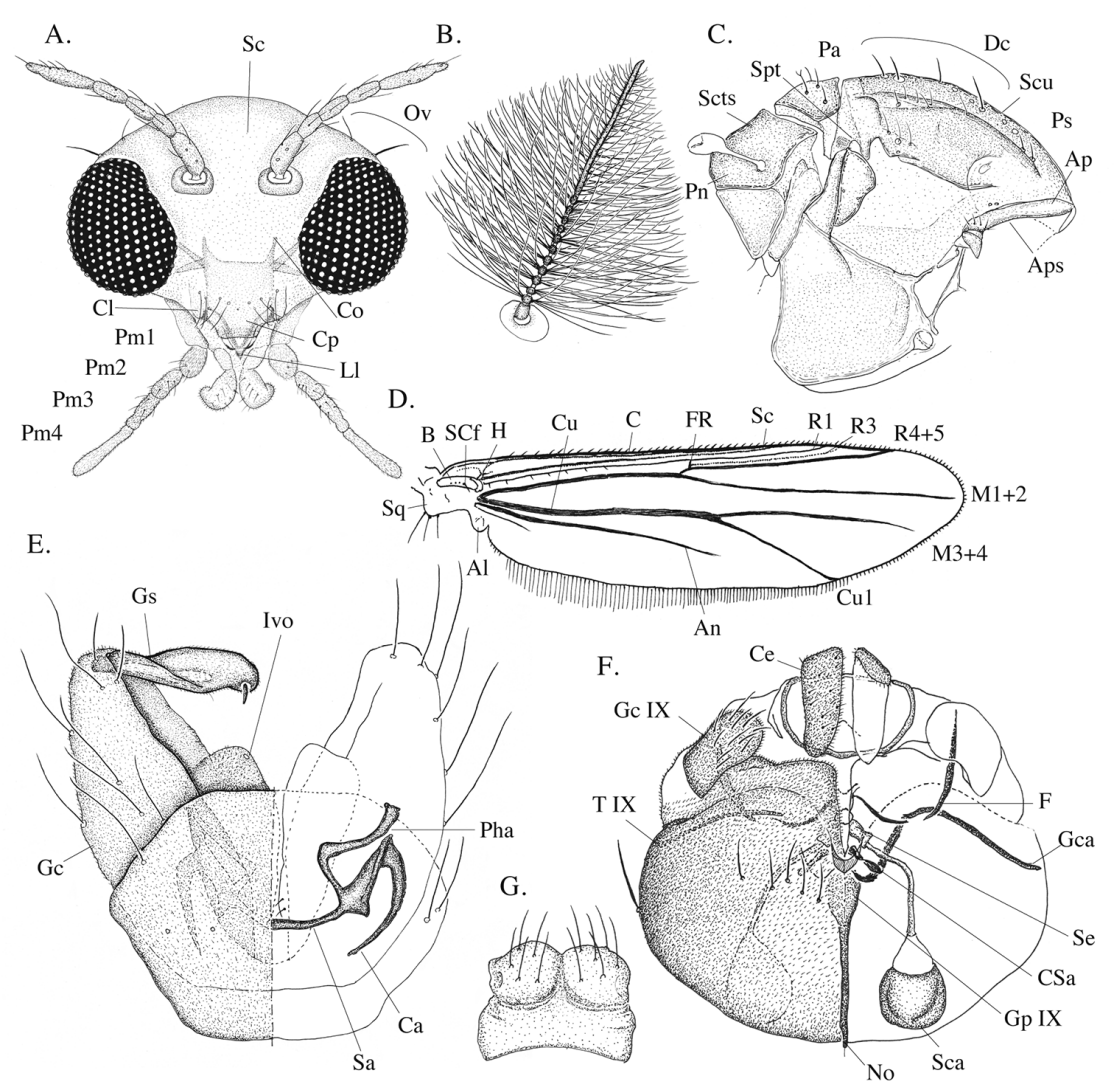
*Eukiefferiella endobryonia* sp. nov., adult. **A** Female head **B** male antenna **C** thorax **D** right wing **E** hypopygium with tergite IX and with left gonocoxite and gonostylus, in dorsal view with gonostylus (left) and in ventral view without gonostylus (right) **F** female genitalia, dorsal (left) and ventral view (right) **G** female tergum IX. Abbreviations (adult). Al: alula; An: anal vein; Ap: antepronotum; Aps: antepronotals; B: brachiolum; C: costa; Ca: coxapodeme; Ce: cercus; Cl: clypeus; Co: cornua; Cp: cibarial pump; Csa: coxosternapodeme; Dc: dorsocentrals; F: fulcrum; Gc: gonocoxite; Gca: gonocozapodeme; Gc IX: gonocoxite IX; Gp IX: gonapophysis IX; Gs: gonostylus; H: humerals; Ivo: inferior volsella; Ll: labial lonchus; No: notum; Pa: prealars; Pha: phallapodeme; Pm: palpal segments; Pn: postnotum; Ps: pseudospurs; Sa: sternapodeme; Sc: subcosta; Sca: seminal capsule; Scts: scutellars; Scu: scutum; Se: spermathecal eminence; Spt: scopula thoracalis; Sq: squama; T IX: tergum IX.

#### Egg.

Unknown.

#### First instar larva.

Unknown.

#### Fourth instar larva.

(*N* = 4) Body length 3.0 mm. Head capsule dark brown. Body yellowish. Head capsule with frontoclypeal apotome with clypeus without divided by strong suture. Antenna nonretractile, 5-segmented; fourth segment twice as long as third segment; lauterborn organ small; blade as long as flagellum; ring organ in basal third. Premandible with one broad, blunt apical tooth. Mandible with apical tooth longer than first lateral tooth; inner margin smooth, without serrations; seta subdentalis short, peg-like; five very long seta interna with five branches divided nearly to the base, each branch similar in length and width to each other; mola with four long spines. Maxilla without pecten galearis; chaetulae of palpiger lacking; lamellae of galea short; anterior lacinial chaeta apparently short, broad-based, more or less differentiated from other chaetae. Mentum with single median tooth and four pairs of lateral teeth; ventromental plates inconspicuous, without beard beneath. Parapods well developed. Claws of anterior parapods all smooth. Procercus unsclerotized, less than 1.5 times as long as wide, without tooth, spur, or seta; anal setae 5–7. Supraanal seta absent. Anal tubules developed, longer than posterior parapods. Body setae very short and inconspicuous, shorter than one-quarter the length of abdominal segments.

#### Pupa.

(*N* = 8) Frontal apotome without frontal seta and warts. Thoracic horn and precorneal seta absent. Dorsocentrals four. Thorax nearly smooth. Wing sheath smooth, without pearl row. T I–II, T VIII, S I and S VIII without shagreen. T II–IX with strong anterior shagreen. S II–VII with weak posterior shagreen. Pedes spurii A and B absent. Caudal spines absent on T II–VIII. S IV–VII female at most with very weak caudal spines. Orally curved hooklets present in uninterrupted rows posterior to caudal spines on T III–V. Apophyses and O setae absent. Segments IV–VIII with very short and weak L-setae. Anal lobe with three unequal anal macrosetae, consisting of two, thinner inner macrosetae and a normal outer macroseta; without median seta, fringe, apical spine.

#### Adult male.

(*N* = 3, if not mentioned) Body length 2.9–3.0 mm without antenna. Body color dark brown. Antennal length 0.8 mm. Flagellum plumose, with 13 flagellomeres; apex spatula-shaped, without a strong straight seta; antennal groove in male reaching flagellomere 3; AR 1.1. Eye bare. Temporal setae 2, not clearly separated into inner and outer verticals and postorbitals. Postocular setae present in a single row, only behind eyes. Palpus 5-segmented; palpomere lengths: 55–72, 86–90, 96, 159–159 (*N* = 1); palpomeres with 3, 4, 5, 0 setae, respectively. Antepronotum well developed with lobes meeting medially at anterior margin of scutum; dorsal anterpronotals absent; four lateral antepronotals; acrostichals absent; six dorsocentrals in a single row. Approximately three prealars. Scutellum smooth with nine scutellars in single row. Supraalar setae present. Wing length 2.3 mm; L/WR 3.01. Wing membrane glabrous, unmarked. Anal lobe small. Costa not extended. Crossvein m-cu absent. Cu_1_ straight. R_4+5_ only fused with C at apex. R_2+3_ present, ending at middle of distance between R_1_ and R_4+5_. Cu_1_ very slightly curved apically at wing margin. Squama with two or three setae. Sensilla campaniformia ca. eight at base of brachiolum, three above setae and eight at apex of brachiolum; 1 on Sc, one basally on R, one near base of R1; and one on FR. Calypter without marginal setae; calyptral fringe absent. First tarsomere of foreleg shorter than fore tibia. Fore coxa not enlarged. Hind tibial comb and tibial spurs reduced; outer spur absent. Pulvilli very faint. Gonostylus hinged to gonocoxite and folded inward. Anal point absent. Anterior margin of transverse sternapodeme convex, phallapodeme and aedeagal lobe normal. Virga absent. Gonocoxite with well-developed inferior volsella. Gonostylus with crista dorsalis; apical spine absent. HR 1.99. Lengths of leg segments and leg ratios as in Table 2.

**Figure 2.**
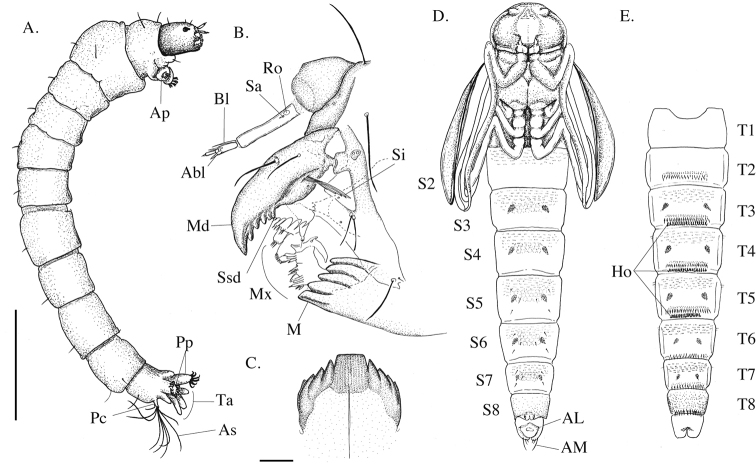
*Eukiefferiella endobryonia* sp. nov., fourth-instar larva and pupa. Larva (**A–C**): **A** general appearance of larva **B** larval antenna, maxilla and mandible, lateral view **C** mentum. Pupa (**D, E**): **D** pupa, ventral aspect **E** ditto, dorsal aspect. Abbreviations (larva). Abl: accessory blade; Ap: anterior parapods; As: anal seta; Bl: blade; M: mentum; Mx: maxilla; Pc: procercus; Pm: premandible; Pp: posterior parapods; Ro: ring organ; Sa: supraanal seta; Si: seta interna; Ssd: seta subdentalis; Ta: anal tubules. Abbreviations (pupa). Al: anal lobe; Am: anal macroseta; Ho: orally curved hooklets. Scale bars: 0.1 mm (**C**), 1 mm (**A**).

**Table 2.** Leg segment lengths of adult male specimens of *E. endobryonia* sp. nov. Data are provided in µm (N = 1). Abbreviations. Fe: femur; Ti: tibia; Ta1-5: tarsal segments 1-5; P1-3: front, mid and hind legs, respectively.

	Fe	Ti	Ta1	Ta2	Ta3	Ta4	Ta5	LR	BV	SV
P1	737.01	675.68	538.6	300.14	216.45	98.12	113.63	0.79	2.67	1.33
P2	831.89	788.23	427.12	292.56	218.61	110.38	124.81	0.54	2.74	1.72
P3	736.29	736.29	540.4	200.21	180.73	91.99	104.25	0.73	3.48	1.59

#### Adult female.

(*N* = 3, if not mentioned) Body length 2.8 mm. Antenna with five flagellomeres; flagellomere lengths (in µm): 56.7, 35.8, 38.2, 45.2, 101.2; with 2, 3, 2, 3, 3 setae, respectively (*N* = 1). Eye bare. Clypeus with 8 setae. R with two setae, squama with 4–6 setae. Scutellum as in male. Gonocoxapodemes not jointed mesally, well sclerotized. Gonocoxite long, with long and short setae. Tergite IX with two unseparated distinct lobes. Triangular floor under vagina present. Gonapophysis VIII pointed caudally, with two apodeme lobe. Membrane T-shaped. Labia small, bluntly quadrangular, void of microtrichia. Seminal capsule ovoid, darker sclerotized in oral half, without microtrichia. Spermathecal ducts with triangular bulb before separate openings. Cercus normal, length twice as long as width.

#### Distribution.

North America (US: Tennessee, Virginia).

#### Etymology.

The species name is a compound word in which three words from Ancient Greek are combined, *endo*- (ἔνδον), a prefix meaning within, *bryon* (βρύον), meaning moss, and the suffix -*ia* (-ία), forming abstract nouns of feminine gender. It alludes to the biology of this species, which live within the case made of mosses.

#### Remarks.

This species is unique among species of *Eukiefferiella* in that its pupae lack the precorneal seta. This species can also be distinguished from others in the genus by the following combination of traits: pupa lacks respiratory horns, and has the unique configuration of pupal anal macrosetae (two thinner inner macrosetae, a normal outer macroseta); and larva has a mentum with four pairs of lateral teeth and a single, wide, and truncate median tooth. Any geographic variation in this species’ characters was detectable between the populations sampled in VA and TN.

#### DNA barcoding.

**Figure 3.**
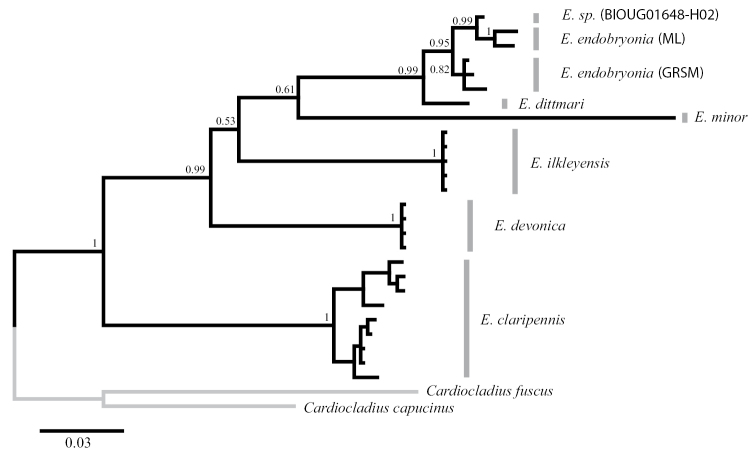
A Bayesian phylogeny based on the COI dataset. Information on the sequences used for this analysis is shown in Table 1. Bayesian Posterior Probabilities were given on each node. Caution is needed to interpret the phylogenetic relationship between and among the members of the species groups (e.g., *devonica* group, consisting of *E. devonica* and *E. ilkleyensis*) included herein, due to the scarcity of available genetic data of the species belonging to the genus *Eukiefferiella*.

The results of the species delimitation analyses are summarized in Table 3. First, BLAST search using the partial COI sequence of voucher YI-CR-001 was executed. This resulted in 98.7 % identical to ‘*Eukiefferiella* sp. voucher BIOUG01648-H02’ in GenBank (accession No. KR660601.1) (Telfer et al. 2015); thus, this sequence was included in the following phylogenetic analyses on the assumption that this specimen may belong to *E. endobryonia* sp. nov. (Table 1). Second, the intra- and inter-specific genetic differentiations were estimated using Bayesian inferences, with the dataset for 28 OTUs. Monophyly of each five species of *Eukiefferiella* was recovered in the Bayesian phylogeny (Fig. 3), as well as *E. endobryonia* sp. nov. (95 % BPP) together with the above-mentioned sequence data. A Bayesian tree indicated that *E. endobryonia* sp. nov. was sister to *E. dittmari* among four species of *Eukiefferiella* in the dataset with significantly high BPP support (Fig. 3). Values of P ID(Strict) for *E. endobryonia* sp. nov. moderately supported the prediction that this species is correctly identified based on the COI sequence (Table 3). Likewise, P(RD) value exceeded 0.05 and hence the clade distinctiveness was supported (Table 3). However, Rosenberg’s P_AB_ value was not significant (P = 0.05) and thus the reciprocal monophyly of the clade of *E. endobryonia* sp. nov. was not supported. Two geographic populations sampled, Great Smoky Mountains (GRSM) and Mountain Lake (ML), formed separate clades and between which genetic divergence among population was substantial (Intra/Inter = 0.12) (Table 3), of which values were equivalent to those of the species clade of *E. claripennis*, composed by the specimens from Europe and Canada (Table 3).

**Table 3.** Summary of the results of species delimitation analysis based on COI. Measures of phylogenetic support and diagnosability of species calculated by species delimitation plug-in in Geneious bioinformatics software are summarized for the species of the genus *Eukiefferiella* included in the dataset, as well as two geographic populations (GRSM and ML) of *E. endobryonia* sp. nov. Monophyly (‘Mono’) was supported for all species/populations. For the other measures, see Materials and methods.

Species	Closest species	Mono	D Intra	D Inter	Intra/Inter	P ID(Strict)	P ID(Liberal)	Av(MRCA)	P(RD)	Rosenberg’s P_AB_
*E. ilkleyensis*	*E. devonica*	yes	0.002	0.152	0.01	0.93 (0.80, 1.0)	0.98 (0.88, 1.0)	9.33E-04	0.05	1.20E-04
*E. devonica*	*E. ilkleyensis*	yes	0.002	0.152	0.01	0.86 (0.72, 1.0)	0.98 (0.87, 1.0)	9.38E-04	0.05	5.20E-05
*E. claripennis*	*E. devonica*	yes	0.024	0.206	0.12	0.91 (0.83, 1.0)	0.97 (0.92, 1.0)	0.0172	0.05	2.50E-08
*E. endobryonia*	*E. ilkleyensis*	yes	0.02	0.163	0.12	0.85 (0.73, 0.98)	0.97 (0.86, 1.0)	0.0131	0.1	0.05
*E. endobryonia* (GRSM)	*E. endobryonia* (ML)	yes	0.008	0.026	0.3	0.59 (0.41, 0.77)	0.84 (0.69, 0.98)	0.0039	0.05	0.02
*E. endobryonia* (ML)	*E. endobryonia* (GRSM)	yes	0.015	0.026	0.57	0.41 (0.23, 0.59)	0.68 (0.53, 0.83)	0.0096	0.11	0.02

#### Habitat.

**Figure 4.**
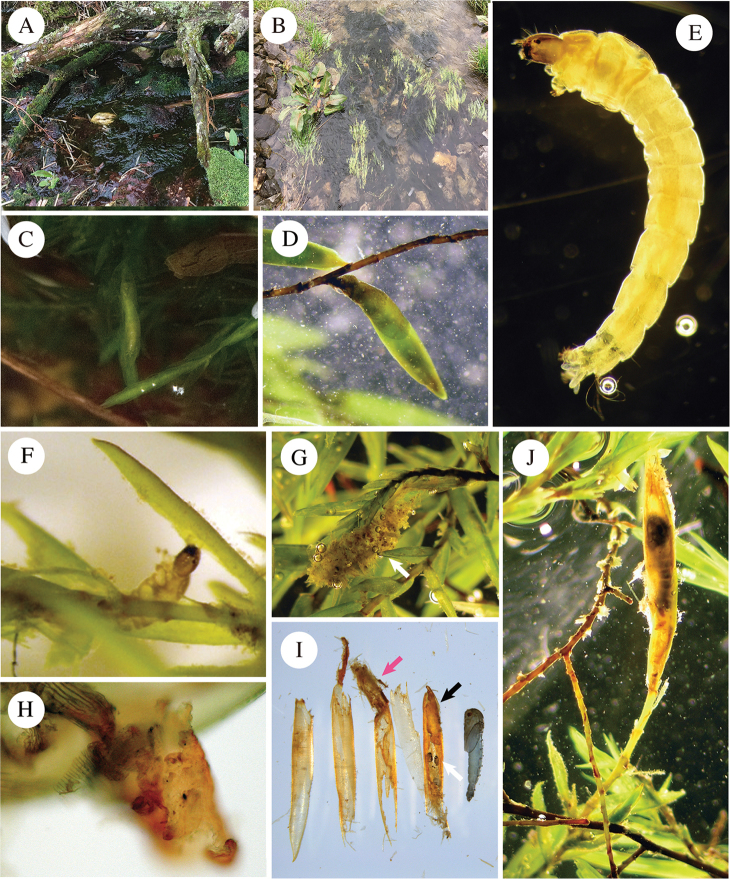
Biology of *Eukiefferiella endobryonia* sp. nov. **A** a colony of *Fontinalis dalecarlica* growing on the sides of pebbles in a gently flowing inlet connected to Mountain Lake, VA, USA (type locality) **B** a colony of *Fontinalis novae-angliae* occurring in a rapidly flowing stream at Sparks Lane, TN, USA **C** early fourth-instar larva, undulating its body in the tube **D** immature capsule of *Fontinalis dalecarlica* attached to the stem underwater **E** fourth-instar larva **F** fourth-instar larva feeding on a leaf margin of *F. dalecarlica***G** a tube structure of the third-instar larva, which was mainly built from particles from the feces of mature larvae **H** amorphous, jelly-like silk mass spotted with detritus and diatoms, ripped off of the inner wall of the inner end of the pupal case **I** a dissected leaf-rolling case, consisting of five leaves and the resident pupa; the leaves used as the case materials are placed in the order of leaf arrangement, with the outermost leaf at the left-most; the innermost leaf (right next to the pupa) contains the head capsule (white arrow) and exuvium of the fourth-instar larva; some debris (pink arrow) and silk mass (black arrow) stuffed in at both ends can be seen **J** a pupa in its case: the pupal head is oriented toward the distal end of the shoot tip; most of the leaves near the pupal case were consumed by the larva early in the fourth instar stage.

Larvae of this species occupied slightly different microhabitats in Mountain Lake, VA (Fig. 4A) and Sparks Lane, TN (Fig. 4B). At Mountain Lake, they inhabited a shallow inlet brook flowing into the sink water of the lake. Some leafy and thallose liverworts, including *Chiloscyphus* (Jungermanniales: Geocalycaceae), as well as some pleurocarpous moss species, such as *Brachythecium* spp. (Hypnales: Brachytheciaceae), were abundant there on the upper sides of boulders and cobbles that were exposed to spray and occasionally submerged in water. *Fontinalis dalecarlica*, a host plant species of *E. endobryonia* sp. nov., occurred at high densities on the lateral sides of submerged boulders in the stream. As a matter of fact, this seemed to be the only aquatic moss species of which conspicuous amounts were found in this particular stream. I was able to find some white-bodied insect larvae occupying some of the apical shoots of *Fontinalis* moss plants (Fig. 4C) simply by looking in the surface layer of slow-moving, shallow water. Interestingly, these larvae apparently resembled the moss capsules enclosed within the bracts of intact plants at first glance (Fig. 4D). At another locality in TN, the larvae occurred in a stream with fast-flowing water. Some clumps of *F. novae-angliae* were found growing in this rapidly flowing stream, which were anchored to the sediment of the streambed. The plants bend 50 cm below the water surface in riffle habitats. Similar to observations in the other population, larval and pupal cases occurred at the terminal ends of moss shoots of *F. novae-angliae*.

#### Life history.

The life cycle of this new species between late spring and early autumn (May–October) is unknown. This species is likely multivoltine because fourth-instar larvae and pupae were found together at both sites in both April and November. It appears that the larvae were collector-gatherers at the third instar, but became scrapers at the fourth instar (*sensu*Berg 1995). The third-instar larva restlessly gathered diatoms, which grew on the rims or surfaces of moss leaves. During the later period of the third larval instar, the larva started to dwell on the apical moss shoots and undulated its body among the terminal leaves. *Fontinalis* leaves are slender, with enrolled margins, and are closely appressed at the tip, forming a semi-enclosed space. At the early stage, a larva showed sinusoidal swimming or undulation behavior (Brackenbury 2000) within the terminal leaves, where it would later make its case. Approximately five days after colonizing the terminal moss leaves, it developed into the fourth larval instar stage (Fig. 4E). The fourth-instar larva seemed incapable of living detached from the case due to its limited locomotory habits. When it was removed from its case, the larva attempted to crawl using the anterior half of its body, but was not able to move forward. It spent most of its time feeding on moss leaves. It extended its head and the anterior part of its body outside of the tube to feed, while using its posterior prolegs to maintain contact with a part of its own tube. It grasped the marginal tissues of moss leaves with its mandibles and dragged them back toward its case (Fig. 4F); simultaneously, silk threads produced from the mouth were extruded with the assistance of the serrated claws of the anterior parapods. The partly grazed leaves were therefore pulled toward the case, which made it easier for the larva to access the surrounding leaf area. The larva repeatedly cut out and fed on the leaves in the bore of the plant in proximity to its case; as a result, ca. 12–20 leaves occurring more or less within ca. 13 mm from the base of the tube were completely consumed (*N* = 6). The foraging areas were therefore mainly restricted to the region immediately surrounding the tube. This territorial feeding behavior has been reported for many tube-dwelling chironomids (e.g., Darby 1962; Jónasson and Kristiansen 1967; Edgar and Meadows 1969; McLachlan 1977, McAuliffe 1984; Leuchs and Neumann 1990). The larva occasionally defecated, and subsequently immediately ejected the fecal pellet from the end of the tube, which is similar to the behavior of *Cladotanytarsus atridorsum* (K.) Edw. (Mackey 1976). The larval fecal pellets were long, ca. half of the body length of the larva, and were loose and cylindrical, which allowed them to easily be released into the water. Under laboratory conditions, younger larvae often used the particles originating from the fecal pellets of mature larvae as tube materials (Fig. 4G), which means these fecal pellets may also be a source of tube material for younger larvae (Hirabayashi and Wotton 1999). Judging from the composition of fragments in the fecal pellets, at this stage, the larva largely relied on moss leaves as a food source, which is supplemented with fine amorphous detritus and epiphytic diatoms. Under laboratory conditions, the larva only occasionally withdrew into the tube and engaged in lateral undulations of the body therein.

The larva became less active in the later period of the fourth instar. It scratched the inner surfaces of the leaf margins, not for consuming the leaves, but presumably for strengthening the case wall. As a result of this intensive fabrication behavior, the tissues of the leaves comprising the case became light brown to red in color due to reactions in the plant tissues, whereas undamaged leaves and stems remained green. Approximately half of the larva’s time was spent spinning silk at this point, and the other half was spent staying still. The spinning behavior was stereotyped, regular, and persisted for more than 5 h at a time. The larva lined the interior of the case with silk, which provided a surface with which the claws of the anal prolegs could engage, anchoring the insect within the case. Due to the fabrication and feeding behavior performed in the earlier stages, there were some apertures in the rolled leaf case on the stem-end side. The larva frequently turned around inside the case to strengthen the case’s inside wall. The innermost leaves in the wall, especially at both ends, thus included a thick layer of silk (Fig. 4H) as a consequence of continuous silk fabrication. Before entering the prepupal stage, debris containing various particles (fecal pellets, diatoms, and strips of moss leaves) was squashed and accumulated at both ends of the case together with a silk mass to seal the end of the tube (Fig. 4I). At the prepupa stage or later, the case consisted of five or six moss leaves, which were firmly enclosed in silk. The larva molted inside the case and casted off the cuticle; the head capsule and exuvium were thus packed into the posterior end of the pupal case (Fig. 4I). Pupation occurred with the head oriented toward the distal end of the case, without exception (*N* = 7) (Fig. 4J). The pupa rested inside the case throughout most of its development. The pupa then swam toward the surface water and emerged as an adult when its development was completed.

## Discussion

### Taxonomic placement of *E. endobryonia* sp. nov.

*Eukiefferiella* represents a large and widespread genus of Orthocladiinae (Moubayed-Breil and Ashe 2013). This genus was erected by Thienemann (1926), and the independence of this genus from others, as well as the validity of its type species, has been debated in many studies (Edwards 1929; Thienemann 1936; Zavřel 1939; Brundin 1956; Lehmann 1972; Sublette and Wirth 1980). This genus is closely related to some orthocladiine genera in the *Cardiocladius* group (Sæther and Halvorsen 1981): *Cardiocladius* Kieff., *Tokunagaia* Sæth., *Tvetenia* Kieff., 1922 (Sæther and Halvorsen 1981), *Nanocladius* Kieff. (Sæther 1977; Lehmann 1972), and *Hydrobaenus* Fries. Keys for European species of this group are available for larvae (Thienemann 1936; Zavřel 1939; Chernovskii 1949; Bode 1983), pupae (Thienemann 1936; Lehmann 1972), adults (Brundin 1956; Lehmann 1972), and all these stages combined (Sæther and Halvorsen 1981).

The study of the taxonomy of *Eukiefferiella* is far from complete. Most existing records from the Nearctic are at the genus, or at most the species-group, level (Epler 2001). Ten species have been recorded from North America (Sæther 1969; Sublette 1970; Simpson and Bode 1980; Oliver et al. 1990; Bode et al. 1996; Patrick 1996; Sandlund and Aagaard 2004; Egan and Ferrington 2015): *E. brevinervis* (Malloch), *E. brevicalcar* (Kieff.), *E. claripennis*, *E. coerulescens* (Kieff.), *E. cyanea* Thienemann, *E. devonica*, *E. gracei* (Edw.), *E. minor*, *E. unicalcar* (Sæth.), and *E. pseudomontana* Goetghebuer. However, there is also evidently a large number of undescribed species (Cranston et al. 1983; Bode 1983; Merritt and Cummins 1996).

*Eukiefferiella endobryonia* sp. nov. was assigned to *Eukiefferiella* on the basis of diagnostic characters proposed by previous studies (Zavřel 1939; Lehmann 1972; Sæther and Halvorsen 1981; Cranston et al. 1983; Bode 1983; Sasa and Kikuchi 1995). Lehmann (1972) suggested that the possession of orally curved hooklets in a row located posteriorly on T III–V is a diagnostic character of the pupae of this genus, although he concluded that there is no single character by which this genus can be defined. Sæther and Halvorsen (1981) emended the definition of *Eukiefferiella* by comparing it to the other genera of the *Cardiocladius* group, and distinguished it based on the following features: lack of a male anal point; presence of a hind tibial comb; female gonapophysis VIII undivided; pupa lacking frontal setae, pearl rows, and a median anal seta; and larvae having a simple S I, seta interna, chaetae, and spinulae. Cranston et al. (1983) noted that *Eukiefferiella* is distinguished from *Tvetenia* based on a combination of the following larval characters: procercus less than 1.5 times as long as wide; abdominal setae shorter than 1/2 the length of an abdominal segment; and a simple S I.

*Eukiefferiella endobryonia* sp. nov. is unique in its lack of a precorneal seta, as most of its congeners have three precorneal setae present in a row or triangle (Lehmann 1972; Coffman et al. 1986); also, the configuration of pupal anal macrosetae (inner two macrosetae reduced, and outer one macroseta regular) is apparently unique for the species of *Eukiefferiella*. The affinity of this new species to others in this genus can thus be problematic. For example, *E. endobryonia* sp. nov. does not key out into any of the species groups proposed by previous authors (Brundin 1956; Lehmann 1972; Cranston et al. 1983; Bode 1983; Coffman et al. 1986). When compared to the species in Japan, this species may have affinity to *chuzeoctava* group defined in Sasa and Kikuchi (1995), although squama has only a few setae in this species (as opposed to squama fringed in the species of *chuzeoctava* group). Some morphological and behavioral traits of *E. endobryonia* sp. nov. show affinity to those of *E. ilkleyensis* in the *E. devonica* group, specifically: the larval mentum medially truncate with four lateral teeth; and the larva of *E. ilkleyensis* is reported to make a case at the shoot tips of the moss *Eurhynchium riparoides* (Hedw.) (Brennan and McLachlan 1980). *E. ilkleyensis* has been recorded from Palaearctic sites (Chernovskii 1949; Brennan and McLachlan 1979; Moller Pillot 2013; Erbaeva and Safronov 2016) but is not known from Nearctic sites. Hence, it is hypothesized that *E. endobryonia* sp. nov. can be included in the *devonica* group; importantly, however, this species morphologically contradicts with the distinguishing characters of the *devonica* group: the pupa of this species does not possess either median antepronotal setae or the row of hooks at the posterior edge of sternite VII (Lehmann 1972); deeply branched seta interna of the larva extending nearly to the base, although shallowly branched seta interna is said to be characteristic of the *E. devonica* group (Epler 2001); further, the larval thoracic setae of the new species are less than one-third as long as the segment from which they arise, as opposed to being more than half as long as the corresponding segment in *E. ilkleyensis* (Chernovskii 1949).

The molecular phylogeny (Fig. 3) clarified that *E. endobryonia* sp. nov. is genetically well-differentiated from *E. ilkleyensis*. This species is suggested to be relatively closely related to *E. dittmari*, which has not been assigned to any species group (Lehmann 1972) but may be incorporated in *devonica* group (Moller Pillot 2013). *E. endobryonia* sp. nov. is morphologically distinguished from *E. dittmari* based on the diagnostic character (i.e., the absence of precorneal setae and unequal length of pupal macrosetae in pupa). The immature stages of *E. dittmari* is not described, but the larvae live in mosses (Moller Pillot 2013). Based on the species delimitation analyses, the species clade of *E. endobryonia* sp. nov. was estimated to be distinctive and a significant level of genetic differentiation is detected between the geographic populations sampled (ML and GRSM). However, Rosenberg’s P_AB_ for each species used herein suggests that the null hypothesis that monophyly of each taxonomic group occurs due to the random coalescent process is not rejected. To clarify the phylogenetic status of the species and to trace the evolutionary history of biology of this group, especially the specialized relationship with mosses, it is necessary to elucidate the biology for the species of which immature stages are unknown and to accumulate morphological and genetic data of the related groups.

### Biology of *Eukiefferiella*

*Eukiefferiella* species are generally abundant in the riffles of rivers and streams (Madder et al. 1977; Brennan and McLachlan 1980; Egan et al. 2014), as well as in the littoral zones of lakes (Thienemann 1936). The species of this genus show a broad spectrum of life histories. Brennan and Mclachlan (1980) suggested that the larval feeding or tube-dwelling behaviors of species in this group may be affected by their habitats (i.e., riffle zones), as these are locations where the supplies of particles and sediments for tube construction are not always abundant.

Several species of *Eukiefferiella* are reported to be ectosymbionts of various freshwater invertebrates, including acting as parasites and commensals (Tokeshi 1995), although in many cases these ectosymbionts are not identified at the species level, or may not even have been described yet. An unidentified species in western North America makes silken tubes within the cases of the caddisfly *Brachycentrus occidentalis* Banks (Trichoptera: Brachycentridae), which may result in a higher mortality rate of the host (Gallepp 1974). *E. aneyla* Svensson and *E. brulini* Moubayed-Breil & Ashe are commensal on limpets of the genus *Ancylus* Müller (Gastropoda: Ancylidae) (Svensson 1986; Moubayed-Breil and Ashe 2013; Schiffels 2014; Fig. 4E). This genus also includes some species that are commensal on naucorid beetles (Roback 1977; Fig. 4H) or mayflies (Winterbourn 2004). Some species use rocks as substrates, including: *E. verrallii* (Edw.) and *E. calvescens* (Edw.), which construct loose tubes incorporating rocky particles (Brennan and McLachlan 1980); and *E. clypeata* Thienemann, which inhabits the exposed regions of rocks swept by water currents and builds solid sheets of silk (Brennan and McLachlan 1980) or cobweb-like tubes (Thienemann 1936; Chernovskii 1949) on the surfaces of stones, similarly to the filter net of hydrospsychid caddisflies. Cocoon-making is only known in one species, *E. claripennis* (Madder et al. 1977).

The larvae of *Eukiefferiella* are frequently found among aquatic mosses, including *Fontinalis* spp. (Zavřel 1939; Bode 1983; Suren 1993; Pinder 1995; Callisto et al. 2007). Interestingly, the fourth-instar larvae and pupae of *E. ilkleyensis* build tubes among the terminal leaves of the long-beaked water feathermoss *Eurhynchium riparoides* (Hedw.) (Hypnales: Brachytheciaceae) (Brennan and McLachlan 1980). *E. ilkleyensis* previously showed a preference for *Fontinalis* moss leaves as case materials (Brennan and McLachlan 1979). Feeding on living and decomposing leaves has been reported for some species (e.g., Callisto et al. 2007). For example, *E. ilkleyensis* is facultatively phytophagous on *Ranunculus calcareus* Butcher (Ranunculaceae) in its later larval instars (Berg 1995; Pinder 1995). The moss-feeding and leaf-rolling behaviors of *E. endobryonia* sp. nov. are apparently related to this species’ biological background. Some morphological traits of this species may be associated with their biology, including the secondary loss of the respiratory horns in the pupa. Respiratory horns represent a major morphological adaptation to oxygen-poor environments (Marziali et al. 2006; Marziali and Rossaro 2006), and tubicolous species often have variously shaped respiratory organs that lack a plastron plate (Cranston 1995). Therefore, the pupa of *E. endobryonia* sp. nov. lacks these additional respiratory adaptations and is dependent on cuticular respiration, although it should be noted that the tubicolous habit itself can be advantageous to respiration (Milne 1938; Walshe 1950; Williams et al. 1987; Kon and Hidaka 1983; Stief et al. 2005).

### Examining the diversity of chironomid tube morphotypes

Building diverse, elaborate tubes is a characteristic behavior of the larvae of Chironomidae. Tube morphology is determined by construction behaviors, which are stereotypical for specific lineages (Chaloner and Wotton 1996; Charbonneau and Hare 1998). Tube construction behavior can therefore be seen as an example of these species’ “extended phenotype” (Dawkins 1982). Importantly, spinning in many chironomids is tightly linked with their feeding activity (Wallace and Merritt 1980; Dudgeon 1994; McLachlan and Ladle 2009). In addition, the case morphology of a species is determined by its habitat and the space, substrata, and particle sources available therein (McLachlan and Cantrell 1976; McLachlan 1977; McLachlan et al. 1978; Brennan and McLachlan 1979; Pinder 1986). However, the features of tubes that result from taxon-specific construction behaviors have often been studied with more emphasis on their food types and functional feeding group (Pinder 1986; Berg 1995; Ferrington et al. 2009). Virtually no previous study has ever given a comprehensive account of the diversity of chironomid tube morphology. Herein, I provide a provisional classification of chironomid tube structures based on their morphotypes (see below), with a summary of species’ biology and some examples. This enables us to give a brief overview of the biology of this group from the perspective of their construction behaviors to deepen our understanding of their ecology and evolution.

Chironomid tube structures can be categorized broadly based on their transportability and the substratum to which the tube is attached. First, tubes can be divided into those with fixed shelters (i.e., the larva cannot move around with the tube) and transportable cases (i.e., the larva freely moves while carrying the tube). Fixed shelters are much more common than transportable cases. Second, fixed shelters can be categorized into three groups (see below) based on the substratum to which the tube is attached. In fact, chironomids as a whole are able to colonize a broad spectrum of substrates. Third, these tube morphotypes can be further subdivided based on the materials out of which the tube is made, but only if the larva has a preference for certain types of particles (e.g., Brennan and McLachlan 1979; Xue and Ali 1997), and most tend to show low specificity for particular tube materials (Brennan and McLachlan 1979).

Tube structure is proximately influenced by the spinning mechanisms of larvae (Webb et al. 1981; Leuchs and Neumann 1990). The silk formation processes differ within the subfamily Chironominae (Webb et al. 1981; Tönjes 1989; Leuchs and Neumann 1990; Ólafsson 1992). Differences in silk properties exist between two subfamilies (i.e., Orthocladiinae and Chironominae) due to the influence of the striated ventromental plates, which are only present in the Chironominae (Churney 1940; Patrick 1966; Webb et al. 1981; Kullberg 1988). Orthocladiine larvae form separate threads of a soft, jelly-like substance, with which they spin loosely woven tubes that are normally attached to hard substrates (Webb et al. 1981). On the contrary, Chironominae manufacture a harder silk that comprises an amorphic liquid secretion while passing the ventromental plates and then becomes a sticky mass in water (Webb et al. 1981). They normally spin compact tubes, in which sediment particles are embedded in a silk matrix (Webb et al. 1981). In addition, some species can produce silk threads with different diameters (Walshe 1951; Leuchs and Neumann 1990), which may be achieved by shearing forces exerted by the ventromental plates or their tensile powers (Leuchs and Neumann 1990).

**Figure 5.**
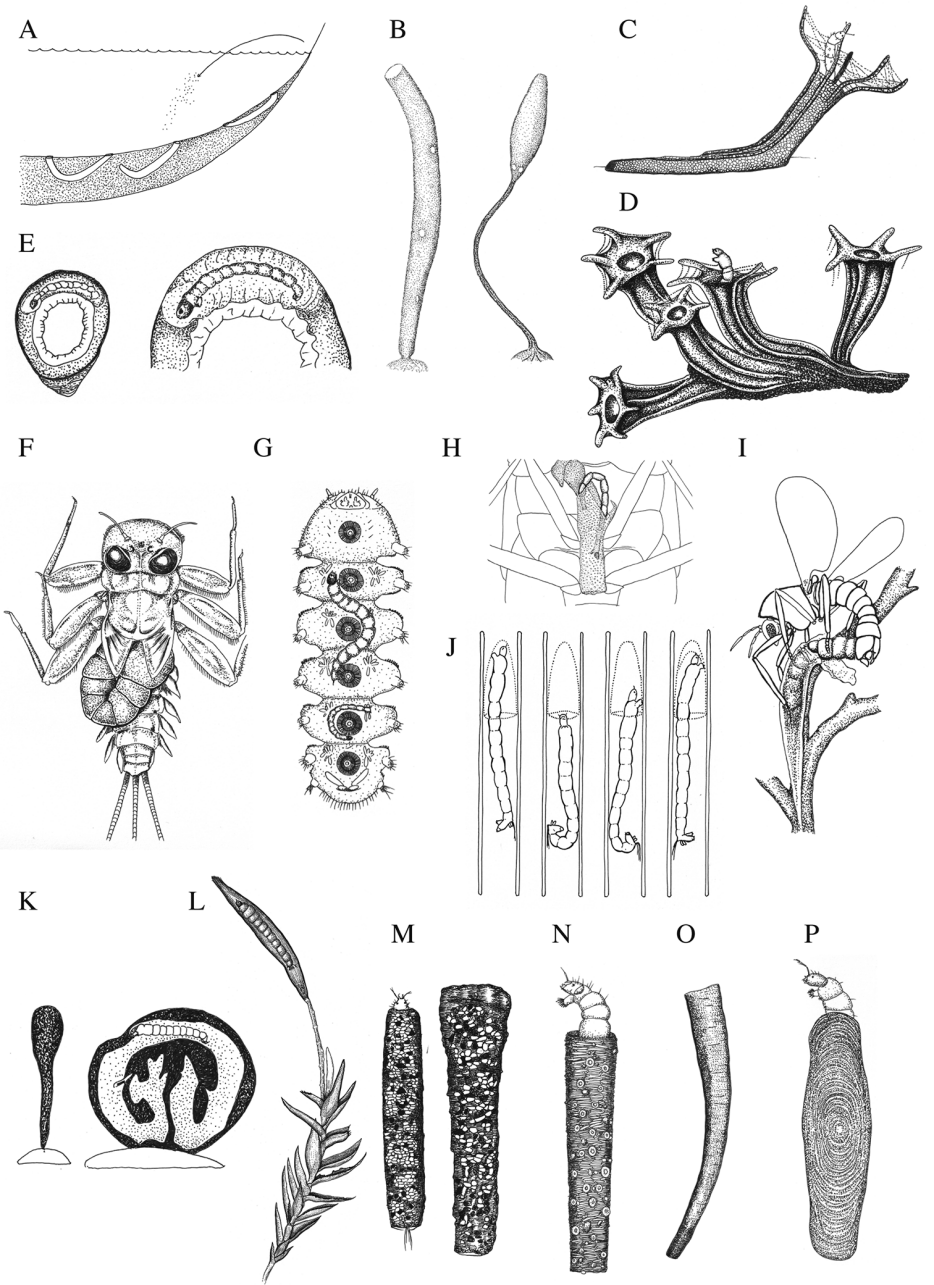
Summary of the biology of chironomids, with special focus on their tube structures. Some taxa without apparent tube structures (**F, G**) are included to give some accounts of their biology. Rock material-bound type (**A–D**): **A** three types of tubes, U-shaped (left), J-shaped (middle), and open-ended (right), built by the mud-dwelling species *Chironomus plumosus*, redrawn from McLachlan and Cantrell (1976)**B** larval (left) and pupal (right) tubes of Orthocladius (Euorthocladius) rivulorum, redrawn from Taylor (1903). **C***Rheotanytarsus rivulorum*, redrawn from Walshe (1951)**D***Rheotanytarsus reissi*, encrusted to form tufa, redrawn from Burmeister and Reiss (2003). Tubes on symbiotic animals (**E–H**): **E***Eukiefferiella brulini* on *Ancylus fluviatilis*, redrawn from Moubayed-Breil and Ashe (2013)**F***Symbiocladius* sp., an obligate parasite of the heptageniid mayfly *Heptagenia lateralis*, redrawn from Codreanu (1939)**G***Tonnoirocladius commensalis* (Tonnoir), which is commensal to the larvae of the net-winged midge (Blephariceridae), redrawn from Tonnoir (1922)**H** silk tube of a Neotropical species of *Eukiefferiella* phoretically attached to *Cryphocricos peruvianus* De Carlo (Naucoridae), in which the open-ended tube is made between the meso- and meta-thoracic coxae of the host, redrawn from Roback (1977). Tubes on/within plants, algae, cyanobacteria (**I–L**): **I** silk tube made among algae by the marine species *Clunio takahashii*, shown *in copula*, redrawn from Hashimoto (1976)**J** a cone-shaped net constructed in the leaf mine of *Endochironomus*, redrawn from Walshe (1951)**K** a tunnel made by *Cricotopus nostocicola* in a spherical colony of cyanobacteria, *Nostoc parmelioides*, redrawn from Brock (1960)**L** a case formed by leaf-rolling on the shoots of *Fontinalis* mosses by *Eukiefferiella endobryonia* sp. nov., based on this study. Portable cases (**M–P**): **M** a solid tube composed of fine detritus constructed by *Stempellinella minor*, redrawn from Brundin (1948)**N** a tubular, firmly constructed case armored with diatoms and rhizopod shells constructed by *Zavrelia pentatoma*, redrawn from Lauterborn (1905)**O** a slightly curved, conifer needle case of *Micropsectra pharetrophora*, redrawn from Fittkau and Reiss (1998)**P** a laterally flattened purse case with concentric growth strips constructed by *Lauterborniella agrayloides*, redrawn from Lauterborn (1905).

Chironomid tubes can most commonly be found bound to rocky materials in virtually all types of streams or lake ecosystems. These represent the first major morphotype of tubes: *(1) rock material-bound tubes*. Rock materials with various grain sizes are used to make tubes, ranging from very coarse (e.g., pebbles, cobbles) to fine (e.g., sand, silt, clay, mud). This type can therefore be subdivided by the grain size of particles used. Soil-dwelling chironomids often occur in lentic or lake environments, where they make soft, cryptic tubes with various lengths and forms, from short, cylindrical tubes to meandering, non-blindly ending tubes, in mud. The shapes of the silk-laden burrows or tubes of species dwelling in soft sediments are associated with their feeding strategies, as these are often deposit- and filter-feeding animals (McLachlan and Cantrell 1976; Leuchs and Neumann 1990; Stief et al. 2005). For example, a mud-dwelling species, *Chironomus plumosus* (Linn.) (Chironominae: Chironomini) (Fig. 5A), makes tubes with a variety of forms, including U- or J-shaped tubes, or horizontal tubes (e.g., McLachlan and Cantrell 1976; McLachlan 1977; LeSage and Harrison 1980; Hodkinson and Williams 1980).

It is noteworthy that three different spinning behaviors are known for species belonging to the genus *Chironomus*, which correspond to the different feeding strategies used by these species. The first method is found in the filter-feeding *C. plumosus*: the larva spins a funnel-shaped net across the lumen of the tube for filtering fine organic particles out of the water (Walshe 1947, 1951). The second method is another filter-feeding strategy, but is more common than the first type: the larva spins a thin layer of silk on the tube wall and then grazes on the food particles that attach to it inside the tube, together with the old net; the tube walls are thus renewed by the larva spinning new bundles of silk threads (Leuchs and Neumann 1990). The third method is deposit-feeding: the larva builds a cylindrical tube and reaches out of their tube to scrape up food particles from the surrounding sediments (Leuchs and Neumann 1990; Stief et al. 2005).

Some species of this type exhibit an aberrant, more elaborate construction behavior than the others. For example, larvae of Orthocladius (Euorthocladius) rivulorum Kieff. (Fig. 5B) make a suspended tube attached by one end to a riffle rock and feed on diatoms growing on the tube (Hershey et al. 1988; Taylor 1903; Peterson et al. 1993). They convert this tube into a pupal tube by binding the basal part with silk to form a long anchoring strand (Taylor 1903), which is similar to those structures made by limnocentropodid caddisflies (Wiggins 2005). The tubes of O. (E.) rivulorum are coated with diatoms, including *Hannaea* Patrick and Reimer (Fragilariaceae), and exert a considerable effect on the periphytic diatom flora (Pringle 1985; Hershey et al. 1988; Herren et al. 2017). Some Tanytarsini (Chironominae) make rigid tubes, such as *Rheotanytarsus* Thienemann & Baus and *Lithotanytarsus* Thienemann. *Rheotanytarsus* (Fig. 5C), which make tubes with arms on which the larvae spin silk strands attached to various substrates (Thienemann 1954; Scott 1967; McLachlan 1977; Kullberg 1988; Spies et al. 2009). *Lithotanytarsus* spp. and *Rheotanytarsus reissi* (Fig. 5D) spin serpentine tubes on the surfaces of travertine rocks, which become encrusted by carbonate mineral deposits, creating tufa over time (Thienemann 1934, 1936, 1954; Pinder 1995; Burmeister and Reiss 2003). Importantly, some ichnofossils have been interpreted as burrowing tubes from lentic environments (Chamberlain 1975; Rodríguez-Aranda and Calvo 1998; Sanz-Montero et al. 2013), with the oldest example of meandering tubes found in Triassic lake deposits (Voigt and Hoppe 2010). Calcareous columnar tufas resembling the biogenic constructions of *Lithotanytarsus* spp. have also been recorded in deposits from the Lower Cretaceous or later (Edwards 1936; Pentecost 2005; Brasier et al. 2011).

Chironomids can also colonize a wide range of organisms, which include motile (animals) or sessile (e.g., plants, cyanobacteria) organisms. Information on the tubes formed by symbiotic chironomids is often limited, and their construction behaviors were not described in many previous studies. The second major group of tubes is: *(2) tubes on symbiotic animals*. Various chironomid lineages (Buchonominae, Podonominae, Chironominae, and Orthocladiinae) exhibit a wide range of symbiotic associations with aquatic animals, ranging from ectoparasitism to phoresis and commensalism, which can be either obligate or facultative associations (Steffan 1968; Tokeshi 1993, 1995; Schiffels 2014). Their hosts are mainly aquatic invertebrates, including snails (Prat et al. 2004; Fig. 5E), caddisflies (Ashe et al. 2015), mayflies (Claassen 1922; Tonnoir 1922; Codreanu 1939; Thienemann 1954; Jacobsen 1995; Fig. 5F), net-winged midges (Cranston 2007; Fig. 5G), naucorid beetles (Fig. 5H), sponges (da Silva Fernandes et al. 2019), and bryozoans (Moller Pillot 2009; Schiffels 2014). Host selectivity and the site of attachment to the host’s body are taxon-specific, and are likely determined by the behavioral patterns of the hosts and the ecological requirements of the chironomids, such as their feeding guilds (Tokeshi 1993). Some species that are tentatively categorized in this group herein presumably do not produce silk (e.g., *Symbiocladius*, Fig. 5F; *Tonnoirocladius*, Fig. 5G). The level of dependency on tube construction seems to differ greatly among taxa. For example, species of *Symbiocladius* (Orthocladiinae) (Fig. 5F) are obligate parasites of mayflies, and their larvae drill under the wing buds of the host and feeds on the host’s hemolymph. *Symbiocladius* spp. are not known to dwell in tubes, but may use other types of attachment mechanisms (Schiffels 2014). Some symbiotic chironomids, particularly facultative symbionts or those that are commensal to the host, tend to make tubes by weaving particles accumulated or flowing in the water on the body surface or shelters of hosts (Schiffels 2014). For example, Winterbourn (2004) gave an account of the tubes of an *Eukiefferiella* sp. in New Zealand, a commensal midge with a mayfly host, throughout its immature stages, and reported that it used silk thread to attach to the host; however, at the third and fourth larval instars, it built an open-ended silken tube above the mayfly’s gills that prevented them from beating, and the wall of this tube incorporated sand grains and fine organic particles.

Similar to the above, the third major category of tubes is: *(3) tubes on/within plants, algae, cyanobacteria.* Some chironomids in the Orthocladiinae and Chironominae often facultatively make tubes on the external surfaces of plants or algae (McGaha 1952; Berg 1995). For example, species of *Telmatogon* Schiner (Telmatogetoninae) inhabit intertidal zones and make their tubes within the green algae occurring on rocks (Cranston 2010). *Clunio* spp. (Orthocladiinae) are marine and make irregular silk tubes dotted with debris on seaweeds, and feed on encrusting diatoms and organic matter (Hashimoto 1976; Archer-Thomson and Cremona 2019). The female adult of *Clunio takahashii* (Fig. 5I) has a degenerated body and copulates with the male without fully extending her body out of her tube, and subsequently lays eggs in the tube (Hashimoto 1976). Many species in Chironominae and Orthocladiinae live inside plant tissues (Berg 1995), mainly as miners, but also rarely as gall-inducers (Jäger-Zürn et al. 2013). Some leaf-mining genera (*Glyptotendipes* Kieff., *Endochironomus* Kieff., and Polypedilum (Pentapedilum) Kieff.) are filter-feeders and spin a conical net inside the mine to filter out floating particles (Fig. 5J), similarly to *Chironomus plumosus* (Washe 1951). Species of *Stenochironomus* Kieff. feed either on living or sequestered plant leaves and tend to show weak preferences for host plants (Kato 2015). *Cricotopus nostocicola* Wirth (Orthocladiinae) (Fig. 5K) has a symbiotic relationship with colonial cyanobacteria in the genus *Nostoc*. The larva makes a tube by feeding on globular colonies of *Nostoc*. The feeding activity of the larva exposes the host to more sunlight, and consequently facilitates photosynthesis by the host (Brock 1960; Dodds and Marra 1989). For many miners (internal-feeders) of plants or algae, silk production has not been confirmed or described. This may be because they are more dependent on their substrates than other taxa. The tube of *E. endobryonia* sp. nov. (Fig. 5L), as described herein, exemplifies one of the few examples of a chironomid that make a tube exclusively using plant materials with silk. This study showed that the larva of this species dwells among the terminal leaves of moss plants, and draws their body out from the tube to consume all of the leaves occurring within a distance reachable by the larva; this species also forms a pupal case consisting solely of the apical five or six leaves of the moss, which are firmly attached together and internally lined by silk. It is interesting that a congener, *E. ilkleyensis*, has been reported to make a tube at the shoot terminus of mosses, yet without using silk for construction (Brennan and McLachlan 1979).

The fourth major group of tubes is: *(4) portable cases*. Among chironomids, transportable cases are far less common than fixed shelters. This is in sharp contrast to the myriad examples of portable cases seen among the Trichoptera (Wiggins 2005). Transportable cases have been found to be made by members of the Chironominae, mainly those in the tribe Tanytarsini, and less commonly in the tribe Chironomini (Walshe 1951; Reiss 1984; Ferrington 1995; Cranston 2010; Stur et al. 2005). *Heterotanytarsus apicalis* (Kieff.) is the only species so far known to the make portable cases among the species in the Orthocladiinae (Moog 1995; Moller Pillot 2013). Many genera of the subtribe Zavreliina in the tribe Tanytarsini, such as *Stempellina* Thienemann & Bause, *Stempellinella* Brundin (Fig. 5M), and *Zavrelia* Kieff. (Fig. 4N), live in cool mountain streams and construct heavy cases with hard shells composed of sand or silt (Lauterborn 1905; Hudson et al. 1990; Stur et al. 2005; Lenz 1924; Ekrem and Stur 2009; Namayandeh et al. 2016). Lauterborn (1905) described a cylindrical case made by *Zavrelia pentatoma* Kieffer & Bause (Fig. 5N), the surface of which was armored with diatoms and rhizopod shells; this species can also build cases exclusively out of rocks (Thienemann 1954). In the subtribe Tanytarsina (Tanytarsini), *Calopsectra* Kieff. and *Micropsectra pharetrophora* Fittkau & Reiss (Chironominae: Tanytarsini) (Fig. 5O) build ‘conifer-needle’ shaped case with spirally arranged sand grains, silt, and long pieces of detritus, especially diatom frustules (Fittkau and Reiss 1998). A few genera of Chironomini, such as *Lauterborniella* Thienemann & Bause and *Zavreliella* Kieff., also make motile cases with sand or silt particles (Thienemann 1954; Moller Pillot 2009). *Lauterborniella agrayloides* (Kieff.) (Fig. 5P) makes a soft purse- or spindle-shaped case that is slightly widened in the middle, strongly pressed together laterally, and rounded at both ends; it also contains characteristic concentric growth strips composed of numerous materials. This case resembles those of hydroptilid caddisflies (Lauterborn 1905). The construction behaviors and preferences for case materials of chironomids that make motile cases have largely not been investigated.

As seen above, many chironomid tubes are comparable to those of caddisflies. Not only are the forms of portable cases made by caddisflies and chironomids similar, but there are also notable shared characters of the pupal cases, known as ‘silken closures’ in caddisflies, between these groups (Wiggins 2005). Diverse forms of silken closures are known among many species of caddisflies in the suborder Integripalpia (Wiggins 2005). These silken closures are disc-shaped masses of silk that are used for closing an open end of the case, and are spun by the final-instar larva before entering pupation. Analogous structures, called ‘Verschlußdeckel (closure cap)’ or ‘Vorderdeckel (front cover)’ by Thienemann (1954), were described in some chironomids by previous studies (Lauterborn 1905; Lenz 1924). This represents a remarkable example of convergent evolution in portable case-makers in two distantly related insect clades (chironomids and caddisflies).

Tube morphotypes can be a tool for use in research in the taxonomy, ecology, and evolution of tube-building animals. The morphotypes of tubes should be assessed if they are useful for taxonomy. The tube, as a functional trait of a specific taxonomic group, may also be useful for examining different species’ ecological niches within a community (McGill et al. 2006). From an ecological perspective, tube functions have been and will continue to be an important subject at various scales, ranging from their adaptive significance for tube-dwelling individuals or species (e.g., Walde and Davies 1984; Williams et al. 1987) to their ecological impacts at the community or ecosystem scales (e.g., Ólafsson and Paterson 2004).

Herein, I made the simplistic assumption that the evolution of case-construction behavior in chironomids could be estimated based on the results of a previous molecular phylogenetic study (Cranston et al. 2012), although the phylogeny of this group has historically been contentious. Buchonominae was recovered as the most plesiomorphic subfamily (Murray and Ashe 1985; Cranston et al. 2012), although this contradicted some previous studies’ conclusions (e.g., Sæther 2000). Importantly, *Buchonomyia* spp. (Buchonominae) are ectoparasites of caddisflies, and supposedly do not manipulate silk (Ashe et al. 2015). The early diverging subfamilies identified, including Podonominae and Tanypodinae, are free-living (Moller Pillot 2013). Among extant lineages, Telmatogetoninae is likely to be one of the earliest-diverging clades of case-builders (Cranston et al. 2012). Tube diversity and construction behavior have evidently diversified in two more derived subfamilies, Chironominae and Orthocladiinae, which split during the mid-Jurassic. Interestingly, the spinning mechanism and silk properties are apparently different among the three clades of tube-making chironomids (Leuchs and Neumann 1990). The different ecological settings and evolutionary backgrounds of these groups have led to them developing a wide range of construction behaviors that have not yet been addressed within a geochronological framework. The emergence of two currently dominant and diverse clades of decomposers (i.e., caddisflies and chironomids) is estimated to have occurred in the Early Triassic (Cranston et al. 2012; Malm et al. 2013). Further, it has been suggested that the major clades of case-builders in both clades appeared contemporaneously during or after the Late Jurassic (Malm et al. 2013). The behavioral convergence of these tube-making insects may have been associated with major changes in freshwater ecosystems that occurred through geological time, such as increased allochthonous inputs of coarse plant materials and fine detritus into these systems (Lancaster and Downes 2013). It would be interesting to examine how these clades of ecosystem engineers have contributed to, or been affected by, the drastic changes in the trophic structures of stream and lake ecosystems that occurred in the Mesozoic Era (Lancaster and Downes 2013; Minter et al. 2016; Buatois et al. 2016; Savrda 2019).

The tube morphology of chironomids does indeed show significant diversity. It is likely that lability in silk production, particle and substrate selection, and construction behavior has made it possible for chironomids to use a broad array of ecological niches, as is the case for the Trichoptera (Weaver and Morse 1986; Wiggins and Mackay 1978; Mackay and Wiggins 1979; Wiggins 2005; Morse et al. 2019). Future investigations of the evolution and diversification of construction behaviors in chironomids in relation to changes in available habitat types will be important to improve our understanding of the evolution of aquatic ecosystems.

## Supplementary Material

XML Treatment for Eukiefferiella endobryonia
